# Intense Innate Immune Responses and Severe Metabolic Disorders in Chicken Embryonic Visceral Tissues Caused by Infection with Highly Virulent Newcastle Disease Virus Compared to the Avirulent Virus: A Bioinformatics Analysis

**DOI:** 10.3390/v14050911

**Published:** 2022-04-27

**Authors:** Shanyu Cheng, Xinxin Liu, Jiaqi Mu, Weiwen Yan, Mengjun Wang, Haoran Chai, Yuxin Sha, Shanshan Jiang, Sijie Wang, Yongning Ren, Chao Gao, Zhuang Ding, Tobias Stoeger, Erdene-Ochir Tseren-Ochir, Aleksandar Dodovski, Pastor Alfonso, Claro N. Mingala, Renfu Yin

**Affiliations:** 1 State Key Laboratory for Zoonotic Diseases, Key Laboratory of Zoonosis Research, Ministry of Education, College of Veterinary Medicine, Jilin University, Xi’an Road 5333, Changchun 130062, China; chengsy19@mails.jlu.edu.cn (S.C.); mujq0623@163.com (J.M.); yanww20@mails.jlu.edu.cn (W.Y.); wangmj20@mails.jlu.edu.cn (M.W.); chaihr19@mails.jlu.edu.cn (H.C.); shayx21@mails.jlu.edu.cn (Y.S.); jiangss21@mails.jlu.edu.cn (S.J.); sijie21@mails.jlu.edu.cn (S.W.); renyn21@mails.jlu.edu.cn (Y.R.); gcning89@jlu.edu.cn (C.G.); dingzhuang@jlu.edu.cn (Z.D.); 2 College of Food Science and Engineering, Jilin University, Xi’an Road 5333, Changchun 130062, China; liuxinx@jlu.edu.cn; 3 Institute of Lung Health and Immunity (LHI), Comprehensive Pneumology Center (CPC), Helmholtz Munich, Member of the German Center for Lung Research (DZL), 85764 Munich, Germany; tobias.stoeger@helmholtz-muenchen.de; 4 Department of Infectious Diseases and Microbiology, School of Veterinary Medicine, Mongolian University of Life Sciences, Ulaanbaatar 17024, Mongolia; erkavet@muls.edu.mn; 5 Department for Avian Diseases, Faculty of Veterinary Medicine—Skopje, Ss. Cyril and Methodius University in Skopje, Lazar Pop Trajkov 5–7, 1000 Skopje, North Macedonia; adodovski@fvm.ukim.edu.mk; 6 Epidemiology Group, National Center for Animal and Plant Health (CENSA), World Organization for Animal Health (OIE) Collaborating Center for the Reduction of the Risk of Disaster in Animal Health, Mayabeque, San José de las Lajas 32700, Cuba; 7 Bureau of Animal Industry, 5 Visayas Ave, Diliman, Quezon City 1100, Philippines; 8 Philippine Carabao Center, Nueva Ecija, Science City of Munoz 3120, Philippines; 9 College of Veterinary Science and Medicine, Central Luzon State University, Nueva Ecija, Science City of Munoz 3120, Philippines

**Keywords:** Newcastle disease virus, chicken embryo, RNA-seq, virulence, host innate immune response, metabolism

## Abstract

The highly virulent Newcastle disease virus (NDV) isolates typically result in severe systemic pathological changes and high mortality in Newcastle disease (ND) illness, whereas avirulent or low-virulence NDV strains can cause subclinical disease with no morbidity and even asymptomatic infections in birds. However, understanding the host’s innate immune responses to infection with either a highly virulent strain or an avirulent strain, and how this response may contribute to severe pathological damages and even mortality upon infection with the highly virulent strain, remain limited. Therefore, the differences in epigenetic and pathogenesis mechanisms between the highly virulent and avirulent strains were explored, by transcriptional profiling of chicken embryonic visceral tissues (CEVT), infected with either the highly virulent NA-1 strain or the avirulent vaccine LaSota strain using RNA-seq. In our current paper, severe systemic pathological changes and high mortality were only observed in chicken embryos infected with the highly virulent NA-1 strains, although the propagation of viruses exhibited no differences between NA-1 and LaSota. Furthermore, virulent NA-1 infection caused intense innate immune responses and severe metabolic disorders in chicken EVT at 36 h post-infection (hpi), instead of 24 hpi, based on the bioinformatics analysis results for the differentially expressed genes (DEGs) between NA-1 and LaSota groups. Notably, an acute hyperinflammatory response, characterized by upregulated inflammatory cytokines, an uncontrolled host immune defense with dysregulated innate immune response-related signaling pathways, as well as severe metabolic disorders with the reorganization of host–cell metabolism were involved in the host defense response to the CEVT infected with the highly virulent NA-1 strain compared to the avirulent vaccine LaSota strain. Taken together, these results indicate that not only the host’s uncontrolled immune response itself, but also the metabolic disorders with viruses hijacking host cell metabolism, may contribute to the pathogenesis of the highly virulent strain in ovo.

## 1. Introduction

Newcastle disease virus (NDV), also known as avian orthoavulavirus 1 (AOAV-1) or avian paramyxovirus 1 (APMV-1), is an enveloped single-stranded negative-sense RNA virus of birds from the genus *Orthoavulavirus* belonging to the subfamily *Avulavirinae* of family *Paramyxoviridae*, with a nonsegmented genome of 15 kb containing coding sequences for six genes [1,2,3]. The virus has infected at least 236 bird species tested to date, including domestic and free-living species [1]. Isolates are of a single serotype, but have a broad virulence from avirulent (lentogenic) to intermediate virulent (mesogenic), and highly virulent (velogenic), depending on the severity of the disease that they cause in birds [1]. Highly virulent viruses that cause diseases with severe morbidity are termed Newcastle disease (ND), which poses a major threat to many species of birds and causes severe economic losses for commercial poultry and pet birds [4]. On the contrary, avirulent viruses cause asymptomatic infections in adult birds or mild respiratory suffering and are widely used as live ND vaccines in commercial poultry [1]. Therefore, vaccines with live, avirulent viruses are the most popular and accepted control and prevention approaches for combating ND in the poultry industry worldwide [5]. However, research on the host’s innate immune responses to infection with either a highly virulent strain or an avirulent strain, and how this response may contribute to severe pathological damage (and even mortality) in response to the infection of a highly virulent strain, remains limited.

Specific-pathogen-free (SPF) chicken embryos are widely used as an ideal and broad experimental model for the infection and propagation of multiple viruses, such as NDV, infectious bronchitis virus (IBV), and avian influenza virus (AIV), because of their accessibility, cost-effectiveness, and fast growth [6,7,8,9,10]. Meanwhile, numerous studies suggested that the immune system in birds starts to grow early during embryogenesis, as well as the fact that numerous immune responses are generated in the latter stage of chicken embryos [11,12]. Furthermore, severe inflammatory pathological lesions and high viral titers were presented in 9- to 10-day-old SPF CEVT when the embryonated chicken eggs (ECE) were challenged with various viruses [13,14,15]. Therefore, to better explore the exact differences in epigenetic and pathogenesis mechanisms between the highly virulent and avirulent strains, we established an SPF chicken embryo model infected with either a highly virulent strain NA-1 (a predominant genotype VII NDV strain presented in parts of most of Asia, including China) or an avirulent widely used vaccine strain LaSota (a genotype II NDV strain) for 24 and 36 h post-infection (hpi), and performed RNA-sequencing (RNA-seq) on viral infected CEVT, to obtain their transcriptional profiles. These findings will not only bring on an understanding of differences in infectivity and pathogenesis between highly virulent and avirulent NDV strains, but also be helpful to novel vaccine development and other control strategies.

## 2. Materials and Methods

### 2.1. Embryonated Eggs and Viruses

The SPF chicken embryos were purchased from Jinan SAIS Poultry Company (Shandong, China). The eggs were incubated at 37.9 °C, with 60% ± 5% humidity for 10 days, and then were infected with viruses or mock. Genotype VII NDV virulent strain NA-1 (GenBank No. DQ659677) [16] and genotype II NDV vaccine strain LaSota (GenBank No. AF077761) were propagated in the allantoic cavity of 9–10-day-old SPF ECE and purified directly from the allantoic fluid; then, the HA titers of allantoic fluid were measured by HA standard assays [17] and stored at −80 °C until used again [18].

### 2.2. Virus Infection, Sample Collection and Preparation

In the current work, 88 10-day-old chicken embryos were randomly divided into three groups, including NA-1 (*n* = 32), LaSota (*n* = 32), and mock (*n* = 24). The allantoic cavity of eggs was injected with either NA-1 or LaSota at a single dose of 7 × 10^3^ TCID50 per egg, and eggs were injected with equal volumes of phosphate-buffered saline (PBS) as the mock-infected group. The inoculated chicken embryos continued to incubate at 37.9 °C and were candled every 6 h to detect dead embryos. Every eight chicken embryos from the viral infected group and six chicken embryos from the mock group were randomly selected at 12, 24, 36 and 48 hpi; then, the allantoic fluid and visceral tissues were collected and stored at −80 °C after quick freezing in liquid nitrogen. The hemagglutination assay tests were performed on allantoic fluid harvested from the ECE [17], and their visceral tissues were sent for RNA-seq.

### 2.3. RNA Extraction, cDNA Library Construction and High Throughput Sequencing

Total RNA was extracted using Total RNA Extractor (Trizol) according to the manufacturer’s instructions (Sangon Biotech, Shanghai, China). Subsequently, RNA concentration was detected by Qubit2.0 (ThermoFisher, Waltham, MA, USA; Carlsbad, CA, USA), RNA purity was evaluated by NanoDrop 2000 (ThermoFisher, Waltham, MA, USA; Carlsbad, CA, USA), and the agarose gel electrophoresis was used to detect RNA integrity and genome contamination, and then qualified RNA samples were selected for cDNA library construction.

For RNA-seq, the mRNA in total RNA was enriched by specific binding of oligo (dT) magnetic beads. Then, the mRNA with poly (A) was eluted from the magnetic beads and fragmented into 200–300 bps fragments. Additionally, cDNA was synthesized by reverse transcription of the enriched mRNA fragments with random primers. After adding the adapter to the two segments of the DNA fragment, the obtained fragment was enriched by PCR to obtain the cDNA library. RNA-seq was performed by Sangon Biotech (Shanghai, China) based on an Illumina Hiseq system (Illumina, San Diego, CA, USA).

### 2.4. Bioinformatic Analysis of Sequencing Data

Transcriptome assembly and annotation protocols were provided by Sangon Biotech in China. At first, low-quality reads, readings containing adaptors, and reads containing poly-N > 10% were removed from the raw data according to the manual of Sangon Biotech, then the sequences of clean reads were aligned with the Gallus gallus reference genome using HISAT2 software, followed by gene expression levels quantified as transcripts per million (TPM) values. The differentially expressed (DE) genes (DEGs) in the groups between NA-1 and LaSota were identified by DESeq2, when corrected to *p* < 0.01 and |log2 (Fold change, FC)| > 1. DEGs were subjected to functional enrichment analysis using clusterProfiler, including gene ontology (GO) enrichment analysis [19] and Kyoto Encyclopedia of Genes Genomes (KEGG) pathway analysis [20]. Studying the distribution of DEGs in the annotation function will clarify the expression of sample differences in gene function. Identified KEGG pathways and GO terms with a corrected *p* < 0.05 were regarded as significantly enriched.

### 2.5. Quantitative Real-Time PCR (qPCR)

To validate the results of RNA-seq, qPCR was carried out using the ABI StepOne Real-Time PCR system (Applied Biosystems, Foster City, CA, USA), and the expression levels of six randomly selected DEGs (OASL, CYP3A5, LDHA, MYD88, TGM2, and CCL4) with annotations from statistical analysis of RNA-seq were measured using the Fast Start Universal SYBR Green Master kit (Roche, Basel, Switzerland). The gene *GAPDH* was selected as the reference gene. The relative expression level of each target gene was calculated based on the 2^−ΔΔCt^ relative expression formula [21].

## 3. Results and Discussion

### 3.1. Distinct Biological Characterization of NDV Highly Virulent Strain NA-1 and Avirulent Vaccine Strain LaSota In Ovo

**Figure 1:**
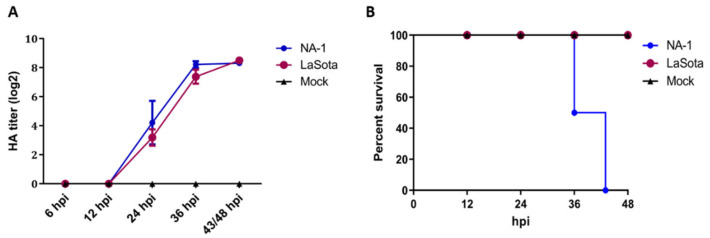
Distinct biological characterization of NDV highly virulent strain NA−1 and avirulent vaccine strain LaSota in ovo. (**A**) Survival percentage of SPF chicken embryos infected with either NDV or mock. The percent survival was observed every 12 h for 48 hpi. (**B**) HA titers were measured in the harvested allantoic fluid of chicken embryos infected with either viruses or mock. The data presented are from three independent experiments, and the result is expressed as the mean ± SEM (standard error of mean). *n* = 6–8.

To investigate the biological properties of different virulence NDV strains, 10-day-old SPF ECE were challenged with a single dose of 7 × 10^3^ TCID50 of either virulent strain NA-1 or avirulent vaccine strain LaSota, and the chicken embryos’ mortality, HA titers of the harvested allantoic fluid and pathological changes in the chicken embryos were evaluated at 12, 24, 36 and 48 hpi, respectively. HA titers of the harvested chicken embryonic allantoic fluid revealed no differences between NA-1 and LaSota groups, although the HA titers at different stages of infection exhibited significant time-dependent increases after 12 hpi which propagated fastest from 12 to 36 hpi (Figure 1A). However, the 10-day-old ECE infected with the virulent strain NA-1 started to appear visible typical hemorrhagic pathological changes in the partly brain and body surface at 24 hpi, and exhibited ecchymosis hemorrhages mainly in the brain and body surface at 36 hpi, while no typical hemorrhagic pathological changes were observed in the brain and body surface of avirulent vaccine strain LaSota group (Figure S1). In addition, the mortality rate reached 50% at 36 hpi and 100% at 43 hpi when the ECE was infected with virulent strain NA-1, while no mortalities were observed following infection with avirulent vaccine strain LaSota and mock (Figure 1B). Collectively, these chicken embryo infection data demonstrated that distinct biological characterizations of different virulence NDV were present in ovo. Therefore, based on these results, the CEVT at 24 and 36 hpi were selected for further RNA-seq.

### 3.2. Global mRNA Expression Patterns in Chicken Embryonic Visceral Tissues after NA-1 and LaSota Infection

**Figure 2:**
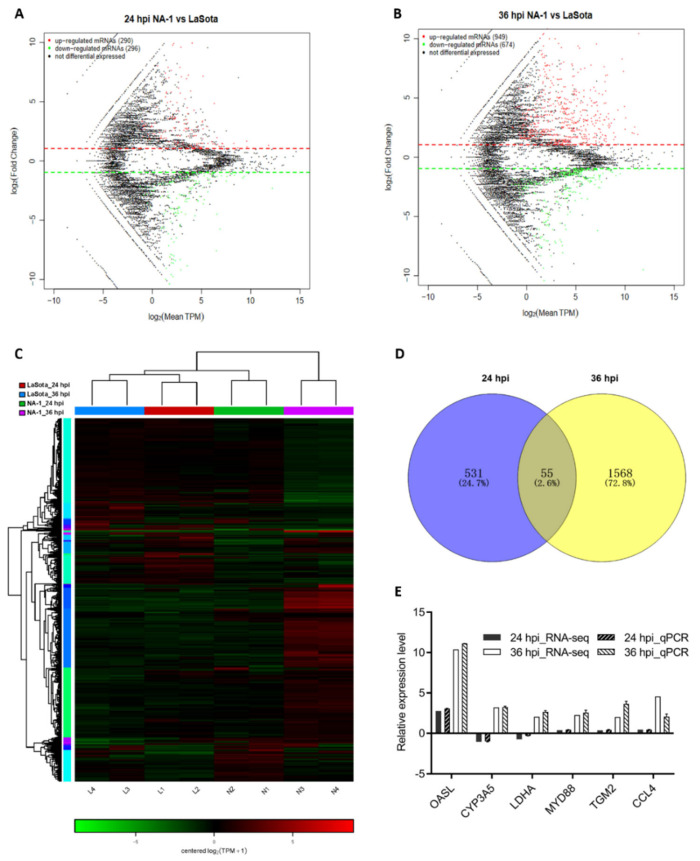
Global mRNA expression patterns in chicken embryonic visceral tissues after NA−1 and LaSota infection. (**A**,**B**) MA plot of DEGs identified in the groups between NA−1 and LaSota at 24 hpi (**A**) and 36 hpi (**B**). The red dots represent upregulated DEGs and the green dots represent downregulated DEGs, respectively. (**C**) Heatmap of DEGs identified in the groups between NA−1 and LaSota at 24 hpi (**A**) and 36 hpi (**B**). Red represents upregulation and green downregulation. (**D**) A Venn diagram of DEGs at 24 and 36 hpi. The blue and yellow circled parts represent the DEGs at 24 hpi and 36 hpi when NA−1 is compared to LaSota, respectively, and the overlapping parts represent the common DEGs in the two comparison groups. (**E**) Verification of the RNA-seq data by quantitative real-time PCR (qPCR). Expression patterns of selected DGEs related to different virulence NDV infections as tested by qPCR. The *y*-axis shows expression levels that are normalized to GAPDH expression. The *x*-axis shows the annotations of the selected DGEs.

To make a thorough inquiry about the exact mechanistic differences behind the above observations between infection by virulent and avirulent NDV strains, a transcriptome sequencing for the CEVT samples was performed. When comparing NA-1 to the LaSota, a total of 2209 DEGs were identified, with 586 (including 290 upregulated and 296 downregulated) and 1623 (including 949 upregulated and 674 downregulated) at 24 and 36 hpi, respectively (Figure 2A–C and Table S1). Among these 2, 209 DEGs, 55 genes were commonly DE at the two-time points, although 531 and 1568 DEGs were uniquely DE at 24 and 36 hpi, respectively (Figure 2D).

**Figure 3:**
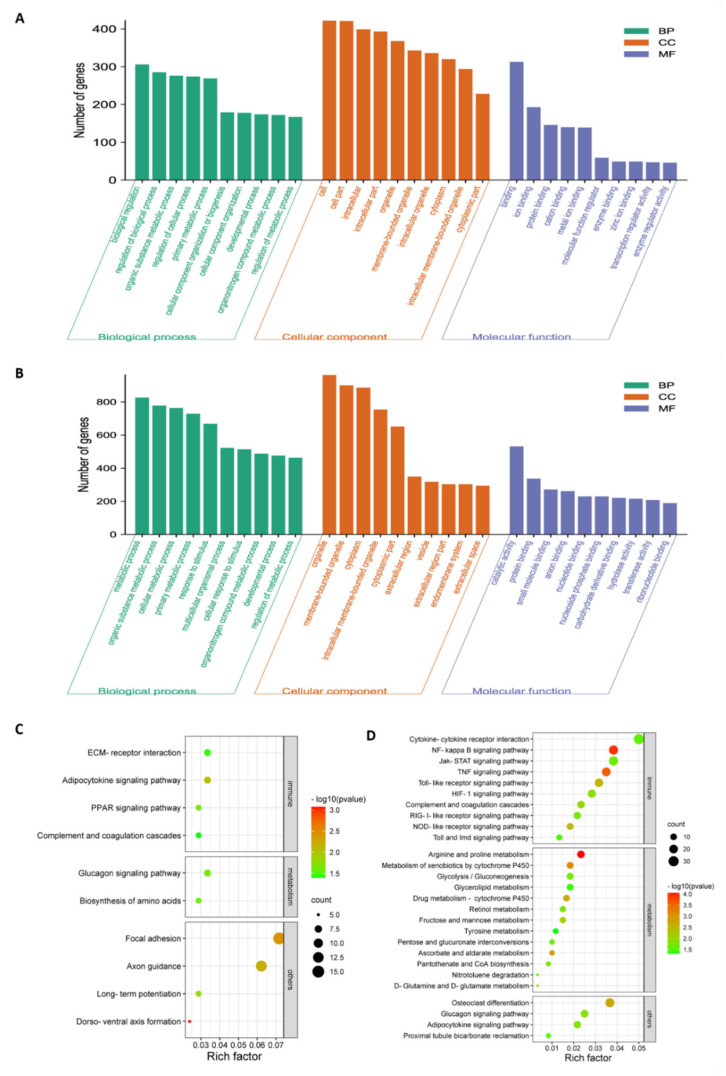
GO and KEGG pathway enrichment analysis of the total DEGs in chicken embryonic visceral tissues infected with viruses. (**A**,**B**) GO enrichment analysis of total DEGs identified in the groups between NA−1 and LaSota at 24 hpi (**A**) and 36 hpi (**B**). The top ten DEGs, according to mean difference (absolute log2FC (fold change)), are presented and sorted by decreasing mean value (*p* < 0.05). (**C**,**D**) KEGG pathway enrichment analysis of total DEGs identified in the groups between NA-1 and LaSota at 24 hpi (**C**) and 36 hpi (**D**). The dot size indicates the number of DEGs. The redder the color, the smaller the *p*-value (*p* < 0.05).

To investigate the potential biological functionalities of the above DEGs, the functional annotation of these DEGs was performed through a GO enrichment analysis (Figure 3A,B and Table S2). When comparing NA-1 to LaSota, the top ten terms within the GO category of biological process that were associated with metabolic processes, such as organic substance metabolic process, primary metabolic process, organonitrogen compound metabolic process, and regulation of metabolic processes, were significantly enriched at 24 and 36 hpi, although two terms named with metabolic process and cellular metabolic process were significantly enriched only at 36 hpi (*p* < 0.05). Meanwhile, common GO terms of cellular components, including organelles, membrane-bounded organelles, cytoplasms, intracellular membrane-bounded organelles, and cytoplasmic parts, were significantly enriched at both time points. Conversely, unique GO terms at 24 and 36 hpi were involved in intracellular, intracellular parts, and intracellular organelles, as well as extracellular regions, extracellular region parts, and extracellular spaces, respectively. Interestingly, molecular functions related to bindings and activities were enriched in completely different ways, as shown in binding, ion binding, protein binding, cation binding, metal ion binding, enzyme binding, zinc ion binding, transcription regulator activity, and enzyme regulator activity. Furthermore, protein binding, small-molecular binding, anion binding, nucleotide binding, nucleoside phosphate binding, carbohydrate-derivative binding, ribonucleotide binding, catalytic activity, hydrolase activity, and transferase activity were significantly enriched at 24 and 36 hpi. Therefore, based on the results from the top ten GO categories of DEGs at these two time points, different enriched GO terms were presented between infection by virulent and avirulent strains, with some common occurrences within three main GO categories (including cellular components, molecular functions, and biological processes), regardless of virulence. Moreover, the unique significantly enriched GO terms during an NA-1 infection relative to those that were identified during LaSota infection could be attributed to differences in epigenetic and pathogenesis mechanisms between the virulent and avirulent strains.

Next, KEGG pathway enrichment analysis was applied as an additional way to elucidate the functions of these DEGs, regardless of upregulation and downregulation. According to the KEGG mapping of the DEGs, 37 significantly enriched KEGG pathways (*p* < 0.05) were obtained during the process of NDV infection at 24 and 36 hpi when comparing NA-1 to LaSota; there were ten at 24 hpi and twenty-seven at 36 hpi (Figure 3C,D and Table S3). Among the 37 KEGG pathways, 10 of the 27 pathways were related to immune response and 13 had metabolism at 36 hpi, but only 1 of the 10 pathways was associated with metabolism and 1 with the immune response at 24 hpi. At the virus infection later stage of 36 hpi, DEGs were enriched in the innate immune response, and inflammatory responses were found to be mainly associated with cytokine–cytokine receptor interaction, the NF-kappa B signaling pathway, Jak-STAT signaling pathway, TNF signaling pathway, Toll-like receptor signaling pathway, HIF-1 signaling pathway, complement and coagulation cascades, RIG-I-like receptor signaling pathway, NOD-like receptor signaling pathway, and Toll and Imd signaling pathway. On the other hand, the enriched pathways were associated with metabolism, including arginine and proline metabolism, metabolism of xenobiotics by cytochrome, ascorbate and aldarate metabolism, drug metabolism—cytochrome P450, D-Glutamine and D-glutamate metabolism, fructose and mannose metabolism, nitrotoluene degradation and five other related pathways. These results, in conjunction with the severe pathological damage observed at a later stage post-infection in ovo, demonstrate that significantly increased enriched pathways related to metabolism and host innate immune responses could contribute to the pathogenesis of the virulent strain in comparison with the avirulent strain.

To further identify the data from RNA-Seq, a subset of six randomly selected genes (OASL, CYP3A5, LDHA, MYD88, TGM2, and CCL4) with annotations from the statistical analysis of RNA-seq was evaluated by qPCR analysis (Figure 2E and Table S4). As shown in Figure 2E, six genes exhibited a concordant direction both in RNA-seq and qPCR analysis. The comparable results indicated that the data obtained by RNA-seq were valid.

### 3.3. Highly Virulent Virus Induces a Robust Innate Immune Response in Chicken Embryonic Visceral Tissues Compared to the Avirulent Vaccine Virus

Previous results demonstrated that infections of virulent NDV elicit strong host innate immune responses in vivo, in vitro, and in ovo, evidenced by the increased expression of inflammatory cytokines, type I and II interferons, IFN effectors, and other host innate immune-related genes and proteins [13,22,23,24]. In Table 1, high expression levels of 25 kinds of inflammatory cytokines and their receptors were induced by the highly virulent strain NA-1 at 36 hpi, including IFNAR1, FLT1, IL10RB, VEGFA, IL8L2, IL13RA1, CSF1, CX3CL1, IL8L1, CRLF2, TNFSF15, IL10RA, TNFRSF9, C-C motif chemokine 26-like, TNFRSF23, TGFBR2, IL17RA, IL1B, TNFRSF10B, CCL4, CSF3, IL15RA, EDA2R, IL6, and CCL19. These findings are consistent with a strong innate immune response to highly virulent NDV after respiratory exposure [22,25]. Among these host factors, the capability of IL-6 to stimulate cytokine production, the migration of macrophages, lymphocytes, dendritic cells, and neutrophils into inflammatory sites [26], as well as the action of IL8L1 and IL8L2 (equivalent to IL8 in humans) to stimulate keratinocytes, endothelial and epithelial cells with the resultant movement of lymphocytes into tissues, indicate their potential roles in viral pathogenesis [27]. Under normal circumstances, these cytokines help coordinate the response of the host immune system against infectious agents [28], such as viruses and bacteria. However, sometimes, the host produces excessive amounts of pro-inflammatory cytokines that trigger inflammation and not enough feedback from the anti-inflammatory cytokines that modulate inflammation, in response to infections of highly virulent influenza virus subtypes, H1N1 and H5N1 [29,30]. Therefore, elevations of several inflammatory cytokines seem to be involved in the development of an acute cytokine storm syndrome, which could be the partial cause of death in chicken embryos infected with highly virulent NDV.

**Figure 4:**
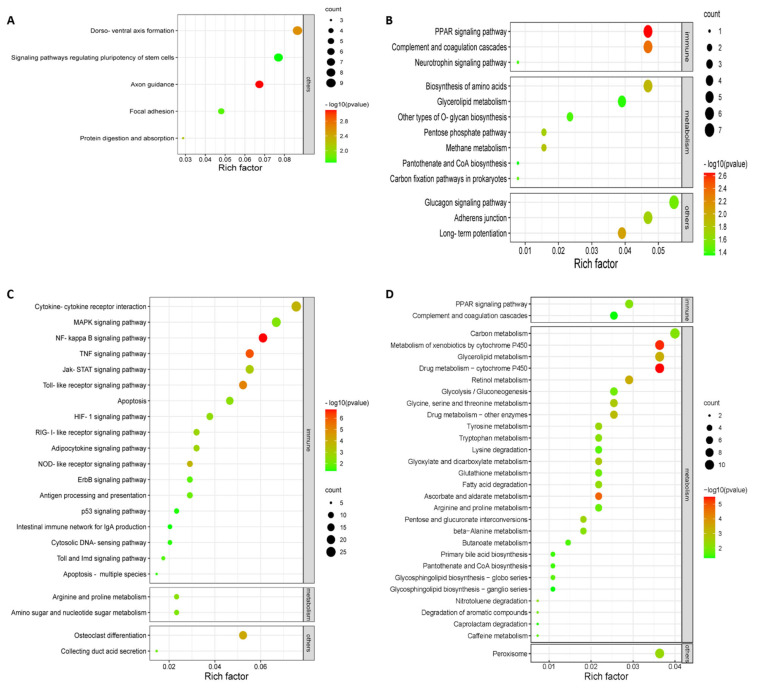
KEGG pathway enrichment analysis for upregulated (**A**,**C**) and downregulated (**B**,**D**) DEGs in the groups of NA−1 vs. LaSota, infected at 24 (**A**,**B**) and 36 (**C**,**D**) hpi, respectively.

Of the upregulated or downregulated DEGs and their enriched KEGG pathways obtained, pathways specifically involved in innate antiviral response, host inflammatory response, and well-defined cytokines were selected for further analysis in this work. According to the KEGG mapping for the upregulated DEGs, a total number of 27 pathways were significantly enriched (*p* < 0.05) with five at 24 hpi and twenty-two at 36 hpi when comparing NA-1 to LaSota (Figure 4A,B), while 43 significantly enriched pathways for the downregulated DEGs were obtained with 13 at 24 hpi and 30 at 36 hpi (Figure 4C,D). Interestingly, when comparing NA-1 to LaSota, 81.82% (18/22) KEGG pathways (enriched by the upregulated DEGs) associated with host innate immune response were induced at 36 hpi, including cytokine–cytokine receptor interaction, the MAPK signaling pathway, NF-kappa B signaling pathway, TNF signaling pathway, and Jak-STAT signaling pathway, whereas no innate immune response-related pathway was enriched at 24 hpi (Figure 4A,B and Table 1). However, 15.15% (5/33) of KEGG pathways related to the host’s innate immune response were enriched based on downregulated DEGs in NA-1 relative to LaSota (Figure 4C,D and Table 1). In addition, numerous upregulated DEGs were specifically involved in the host immune system, including antigen processing and presentation, complement and coagulation cascades, intestinal immune network for IgA production, and the cytosolic DNA-sensing pathway.

Three classes of pattern-recognition receptors (PRRs), designated NOD-like receptors (NLRs), retinoic acid-inducible gene I (RIG-I)-like receptors (RLRs), and Toll-like receptors (TLRs), were demonstrated to be involved in the recognition of pathogen-associated molecular patterns (PAMPs) in various innate immune cells [31,32]. Among these receptor types, RLRs and TLRs are essential for the production of proinflammatory cytokines and type I interferons (IFNs), whereas NLRs are important to regulate interleukin-1β (IL-1β) production [31,32,33]. In the host, the antiviral innate immune response should be of a suitable magnitude and duration to competently eradicate the invading viruses; however, an overactive innate immune response can cause immune pathology and subsequent undesirable damage [34]. Similarly, in our work, when comparing NA-1 to LaSota at 36 hpi, a total of 23 KEGG pathways related to host innate immune response—including three classes of PRRs signaling pathways, the NF-kappa B signaling pathway, and others—were significantly enriched by DEGs (Figure 4). Therefore, we speculate that the infection of highly virulent NDV causes the hyperactivation of various host innate immunity and promotes the pathogenesis of ND illness.

### 3.4. Severe Metabolic Disorders in Chicken Embryonic Visceral Tissues Caused by Highly Virulent Virus Compared to the Avirulent Vaccine Virus

Metabolism consists of numerous interconnected cellular pathways within the cells of living organisms to ultimately provide cells with the energy required to sustain life [35]. Over the last decades, it has been confirmed that viruses dramatically promote cellular anabolism for the generation of the macromolecules needed for virion assembly, viral genome replication, and envelopment of the enveloped viral particles, since they do not innately have their own metabolism [36,37]. Therefore, the recognition of how viruses reprogram cellular metabolism, and where in the virus life cycle these metabolic alterations are required, will present a comprehensive interpretation of virus propagation needs and potentially deliver defense strategies against viral invasions. Several host central cellular metabolic pathways, including lipid metabolism, vitamin metabolism, thymidine metabolism, and amino acid metabolism, are significantly reprogrammed by NDV infection [13,38,39]. However, there is very limited knowledge regarding the recognition of how different virulent NDV reprogram cellular metabolism and whether highly virulent NDV-induced metabolic pathways alterations could be the main cause of severe systemic pathological changes and high-mortality ND illness. According to the KEGG mapping, in total, 36 metabolic pathways were significantly enriched (*p* < 0.05) with 7 at 24 hpi and 29 at 36 hpi when comparing NA-1 to LaSota (Figure 4C,D). With the progress of a highly virulent NDV propagation, the numbers of the reprogrammed cellular metabolic pathways increased; therefore, the results presented here are in line with previous studies [38,40].

Many viruses have been indicated to alter various aspects of host central carbon metabolism, including elevated glycolysis and increased pentose phosphate activity, in order to promote the production of amino acids and nucleotides, as well as lipid synthesis [37,41]. However, in our work, 94.44% (34/36) metabolic pathways were enriched by downregulated DEGs at two time points, whereas only two metabolic pathways (including arginine and proline metabolism, as well as amino sugar and nucleotide sugar metabolism) were enriched by upregulated DEGs at 36 hpi rather than 24 hpi (Table 1). To our surprise, among the 34 metabolic pathways associated with downregulated DEGs, 25 pathways were found at 36 hpi (Figure 4D), a time point with a mortality rate of 50% when the chicken embryo was infected with the highly virulent strain NA-1 (Figure 1B). Together with the different pathological changes observed at 36 hpi in ovo, we speculated that the infection of highly virulent NDV induces a severe imbalance of reprogrammed cellular metabolic pathways between host cell supplies and virus requirements and aggravated the life-threatening symptoms in the infected cell, while avirulent LaSota results in reprogrammed cellular metabolic pathways with a suitable magnitude and duration.

## 4. Conclusions

In conclusion, our study clearly confirms that highly virulent NDV drives robust innate immune responses and severe metabolic disorders to promote the immunopathological damages of ND illness, and thus life-threatening symptoms in severely affected animals. However, more studies concerning the host’s innate immune responses and metabolic disorders are needed to further explore the exact mechanisms involved in the interaction between the host and highly virulent NDV as compared to the avirulent NDV.

## Figures and Tables

**Table 1:** Detailed information about the host’s innate immune response and metabolism-related KEGG pathways enriched by upregulated and downregulated DEGs.

Related Pathways	Upregulated or Downregulated DEGs	NA_1 vs. LaSota_24 hpi	NA_1 vs. LaSota_36 hpi
Host innate immune response	Upregulated	None	(1) TNF signaling pathway (*p* = 0.000000824): PRKCQ, TRIM25, IL8L2, IL8L1, MAP3K14, TNFAIP3, PTGS2, VCAM1, NFKB2, IL1B, CCL4, E3 ubiquitin/ISG15 ligase TRIM25-like, PCASP3, TRAF6, BIRC3, MYD88, CCL19, PLCG1, NFKBIA (2) Jak-STAT signaling pathway (*p* = 0.000804246): IFNAR1, IL10RB, MYC, STAT1, MCL1, CDKN1A, IL13RA1, CISH, STAT3, CRLF2, IL10RA, PIM1, STAT2, PTPN2, SOCS3, SOCS1, CSF3, IL15RA, IL6 (3) Toll and Imd signaling pathway (*p* = 0.020634867): JUN, REL, BIRC3, MYD88, NFKBIA, FBXW11 (4) RIG-I-like receptor signaling pathway (*p* = 0.00174338): DDX3X, IRF7, TRIM25, IL8L2, IL8L1, IFIH1, DHX58, E3 ubiquitin/ISG15 ligase TRIM25-like, TRAF6, AZI2, NFKBIA (5) NOD-like receptor signaling pathway (*p* = 0.000164104): IL8L2, IL8L1, TNFAIP3, RIPK2, IL1B, CARD8, TRAF6, IL6, BIRC3, NFKBIA (6) Toll-like receptor signaling pathway (*p* = 0.000007228): IFNAR1, IRF7, STAT1, TLR3, IL8L2, JUN, IL8L1, SPP1, FOS, IL1B, CCL4, CD80, TRAF6, IL6, MAP3K8, MYD88, NFKBIA (7) HIF-1 signaling pathway (*p* = 0.003034547): FLT1, VEGFA, CDKN1A, ANGPT1, STAT3, ARNT, EDN1, NOS2, PFKFB3, HK2, IL6, SLC2A1, PLCG1 (8) NF-kappa B signaling pathway (*p* = 0.000000172): PRKCQ, TRIM25, IL8L2, IL8L1, MAP3K14, TNFAIP3, PTGS2, VCAM1, C-C motif chemokine 26-like, NFKB2, IL1B, CCL4, E3 ubiquitin/ISG15 ligase TRIM25-like, PCASP3, TRAF6, BIRC3, MYD88, CCL19, PLCG1, NFKBIA (9) Cytokine–cytokine receptor interaction (*p* = 0.000144898): IFNAR1, FLT1, IL10RB, VEGFA, IL8L2, IL13RA1, CSF1, CX3CL1, IL8L1, CRLF2, TNFSF15, IL10RA, TNFRSF9, C-C motif chemokine 26-like, TNFRSF23, TGFBR2, IL17RA, IL1B, TNFRSF10B, CCL4, CSF3, IL15RA, EDA2R, IL6, CCL19 (10) MAPK signaling pathway (*p* = 0.005574722): MYC, GADD45G, DUSP16, MAP3K5, JUN, MAP3K14, DUSP8, JUND, MAP3K2, beta-arrestin-1, TAOK3, TGFBR2, NFKB2, FLNB, FOS, IL1B, HSPA2, TRAF6, DUSP10, MAP3K8, DUSP5, NR4A1, GADD45B (11) Apoptosis (*p* = 0.003742911): GADD45G, PMAIP1, MCL1, MAP3K5, JUN, MAP3K14, TNFRSF23, CTSS, FOS, TNFRSF10B, APAF1, BAK1, CASP7, BIRC3, NFKBIA, GADD45B (12) Antigen processing and presentation (*p* = 0.011647748): RFX5, TAP2, BF2, TAP1, BF1, CTSS, HSPA2, DMB2, B2M, TAPBP (13) ErbB signaling pathway (*p* = 0.018197229): MYC, ABL2, PAK4, CDKN1A, JUN, AREG, SRC, EREG, ERBB3, PLCG1 (14) Apoptosis—multiple species (*p* = 0.033633376): PMAIP1, APAF1, BAK1, CASP7, BIRC3 (15) Cytosolic DNA-sensing pathway (*p* = 0.037686342): IRF7, ADAR, IL1B, CCL4, IL6, NFKBIA (16) p53 signaling pathway (*p* = 0.040178704): GADD45G, PMAIP1, CDKN1A, PPM1D, THBS1, SHISA5, APAF1, GADD45B (17) Intestinal immune network for IgA production (*p* = 0.04499801): MADCAM1, MAP3K14, DMB2, CD80, IL15RA, ICOSLG, IL6
	Downregulated	(1) PPAR signaling pathway (*p* = 0.002276561): GK2, CD36, PPARA, ACOX1, SORBS2, RXRG (2) Complement and coagulation cascades (*p* = 0.004292661): VWF, SERPIND1, CFI, SERPINF2, KNG1, A2M (3) Neurotrophin signaling pathway (*p* = 0.038924743): TRAF6	(1) PPAR signaling pathway (*p* = 0.008314028): ACSL1, SLC27A2, APOA2, CYP8B1, CD36, RXRG, CYP27A1, SLC27A4 (2) Complement and coagulation cascades (*p* = 0.047342556): C5, F7, CFI, F13B, F10, A2ML3, A2M
Metabolism	Upregulated	None	Amino acid metabolism (1) Arginine and proline metabolism (*p* = 0.003997042): CARNS1, ODC, PRODH, P4HA2, SAT1, NOS2, P4HA1, AZIN2 Carbohydrate metabolism (1) Amino sugar and nucleotide sugar metabolism (*p* = 0.006128738): NANP, acidic mammalian chitinase 2, PMM1, UGP2, GNPNAT1, HK2, FUK, GFPT2
	Downregulated	Carbohydrate metabolism (1) Pentose phosphate pathway (*p* = 0.018436337): PFKP, RBK Energy metabolism (1) Methane metabolism (*p* = 0.014671631): PSAT1, ESD (2) Carbon fixation pathways in prokaryotes (*p* = 0.034104959): ACLY Glycan biosynthesis and metabolism (1) Other types of O-glycan biosynthesis (*p* = 0.038361492): POMGNT1, B4GALT1, OGT Lipid metabolism (1) Glycerolipid metabolism (*p* = 0.042424504): AGPAT3, GK2, GPAM, LPIN2 Metabolism of cofactors and vitamins (1) Pantothenate and CoA biosynthesis (*p* = 0.044575194): DPYD Others (1) Biosynthesis of amino acids (*p* = 0.012200804): CBSL, PFKP, IDH1, MAT1A, PSAT1	Amino acid metabolism (1) Glycine, serine and threonine metabolism (*p*= 0.001812138): GATM, GAMT, AGXT, BHMT2, DMGDH, GCSH, GNMT (2) Tyrosine metabolism (*p* = 0.004022975): HGD, ADH6, COMT, FAHD1, ADH1C, ADH1L (3) Tryptophan metabolism (*p* = 0.007340252): KMO, CAT, IDO2, ALDH3A2, ALDH9A1, CYP1B1 (4) Arginine and proline metabolism (*p* = 0.017275567): GATM, GAMT, SRM, PRODH2, ALDH3A2, ALDH9A1 Biosynthesis of other secondary metabolites (1) Caffeine metabolism (*p*= 0.021647064): NAT, PNAT10 Carbohydrate metabolism (1) Ascorbate and aldarate metabolism (*p* = 0.000016731): UGT1A1, L-gulonolactone oxidase-like, ALDH3A2, RGN, ALDH9A1, MIOX (2) Glycerolipid metabolism (*p* = 0.000434007): AGPAT3, TKFC, MGLL, AKR1A1, GPAM, DGAT2, ALDH3A2, ALDH9A1, AWAT1, GLA (3) Glyoxylate and dicarboxylate metabolism (*p*= 0.001971562): HAO1, AGXT, PGP, CAT, HAO2, GCSH (4) Pentose and glucuronate interconversions (*p*= 0.003590513): UGT1A1, AKR1A1, ALDH3A2, SORD, DCXR (5) Glycolysis/gluconeogenesis (*p*= 0.013937081): ADH6, AKR1A1, ALDH3A2, ALDH9A1, GALM, ADH1C, ADH1L Lipid metabolism (1) Fatty acid degradation (*p* = 0.00550051): ACSL1, ADH6, ALDH3A2, ALDH9A1, ADH1C, ADH1L (2) Primary bile acid biosynthesis (*p* = 0.034097227): CYP8B1, AMACR, CYP27A1 Lycan biosynthesis and metabolism (1) Glycosphingolipid biosynthesis—globo series (*p* = 0.023034117): ST3GAL1, ST3GAL2, GLA (2) Glycosphingolipid biosynthesis—ganglio series (*p* = 0.047434041): ST3GAL1, beta-1,3-galactosyltransferase 4-like, ST3GAL2 Metabolism of cofactors and vitamins (1) Retinol metabolism (*p* = 0.000403502): RDH16, ALDH1A1, ADH6, UGT1A1, SDR16C5, RDH10, ADH1C, ADH1L (2) Pantothenate and CoA biosynthesis (*p*= 0.034097227): PPCS, PANK1, DPYS Metabolism of other amino acids (1) beta-Alanine metabolism (*p* = 0.007641542): SRM, ABAT, ALDH3A2, ALDH9A1, DPYS (2) Glutathione metabolism (*p* = 0.015480884): GGT5, GSTAL3, GSTT1, GSTA3, SRM, GSTA2 Xenobiotics biodegradation and metabolism (1) Drug metabolism—cytochrome P450 (*p* = 0.000003098): GSTAL3, GSTT1, ADH6, UGT1A1, GSTA3, FMO4, GSTA2, FMO3, ADH1C, ADH1L (2) Metabolism of xenobiotics by cytochrome P450 (*p* = 0.000004152): EPHX1L, GSTAL3, GSTT1, ADH6, UGT1A1, GSTA3, GSTA2, ADH1C, ADH1L, CYP1B1 (3) Drug metabolism—other enzymes (*p*= 0.000889351): CES1L2, NAT, UGT1A1, thymidine phosphorylase, fatty acyl-CoA hydrolase precursor, medium chain-like, PNAT10, DPYS (4) Nitrotoluene degradation (*p*= 0.006933666): NAT, PNAT10 (5) Caprolactam degradation (*p* = 0.042600529): AKR1A1, RGN Others (1) Carbon metabolism (*p* = 0.008613237): HAO1, AGXT, ESD, PGP, TKFC, GLUD2, CAT, RPIA, H6PD, HAO2, RGN (2) Degradation of aromatic compounds (*p* = 0.013419671): AKR1A1, RGN

## Supplementary Materials

The following supporting information can be downloaded at: https://www.mdpi.com/article/10.3390/v14050911/s1, Figure S1: Pathological changes of the chicken embryos infected with either NDV or mock. The typical hemorrhagic pathological changes were presented in NA-1-infected SPF chicken embryos after 24 hpi, while no pathological changes were observed in both LaSota-infected and mock-infected groups at all time points; Table S1: Detailed list regarding all DEGs in the groups between NA-1 and LaSota at 24 and 36 hpi; Table S2: GO enrichment analysis for all DEGs in the groups between NA-1 and LaSota at 24 and 36 hpi; Table S3: KEGG pathway enrichment analysis for all DEGs in the groups between NA-1 and LaSota at 24 and 36 hpi; Table S4: Primers used in this work.
