# Supplementary material for: Intense Innate Immune Responses and Severe Metabolic Disorders in Chicken Embryonic Visceral Tissues Caused by Infection with Highly Virulent Newcastle Disease Virus Compared to the Avirulent Virus: A Bioinformatics Analysis
Source: Viruses. 2022 Apr 27;14(5):911. doi: 10.3390/v14050911 (PMC9145607; doi:10.3390/v14050911)
Supplement: Supplementary file 1 [file viruses-14-00911-s001.zip › Figure S1.pdf]

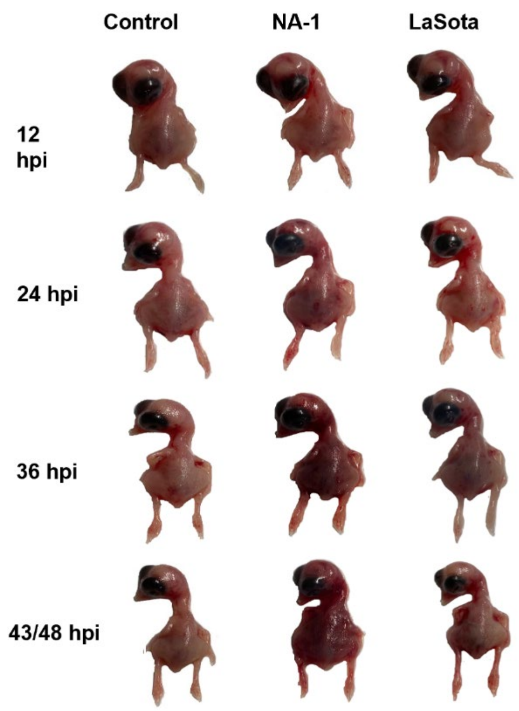

Figure S1. Pathological changes of the chicken embryos infected with either NDV or mock. The typical hemorrhagic pathological changes were presented in NA-1-infected SPF chicken embryos after 24 hpi, while no pathological changes were observed in both LaSota-infected and mock-infected groups at all time points.
