# Supplementary material for: Intense Innate Immune Responses and Severe Metabolic Disorders in Chicken Embryonic Visceral Tissues Caused by Infection with Highly Virulent Newcastle Disease Virus Compared to the Avirulent Virus: A Bioinformatics Analysis
Source: Viruses. 2022 Apr 27;14(5):911. doi: 10.3390/v14050911 (PMC9145607; doi:10.3390/v14050911)
Supplement: Supplementary file 1 [file viruses-14-00911-s001.zip › Table S4.pdf]

Table S4. Primers used in this work.

Gene	primer sequence (5'-3')	reference
OASL	F: AGATGTTGAAGCCGAAGTACCC	[1]
	R: CTGAAGTCCTCCCTGCCTGT	
CYP3A5	F: GTCACAAGCACTTCCTTCG	[2]
	R: AATGACAAGATGAAGACTGGAT	
LDHA	F: AGGACTTGGCAGATGAAC	[2]
	R: AGCAGTGACAATGACCAG	
MYD88	F: TTACGAAGGAAGCAGCAGGA	[3]
	R: CTGACAGTAGCAGATGAAGGCA	
TGM2	F: CACGACACCAACGGCAACC	[1]
	R: GACCTCCGCAAAGACGAA	
CCL4	F: CCTCGCTGTCCTCCTCATT	[3]
	R: CACTGGCTGTTGGTCTCGT	
GAPDH	F: GAGGGTAGTGAAGGCTGCTG	[4]
	R: CACAACACGGTTGCTGTATC	

References:

1. Jia, Y.Q., et al., *Common microRNA(-)mRNA Interactions in Different Newcastle Disease Virus-Infected Chicken Embryonic Visceral Tissues.* Int J Mol Sci, 2018. **19**(5).
2. Fan, X.C., et al., *Genome-wide analysis of differentially expressed profiles of mRNAs, lncRNAs and circRNAs in chickens during Eimeria necatrix infection.* Parasit Vectors, 2020. **13**(1): p. 167.
3. Wu, G., et al., *Cecal MicroRNAome response to Salmonella enterica serovar Enteritidis infection in White Leghorn Layer.* BMC Genomics, 2017. **18**(1): p. 77.
4. Chen, Y., et al., *Gga-miR-19b-3p Inhibits Newcastle Disease Virus Replication by Suppressing Inflammatory Response via Targeting RNF11 and ZMYND11.* Front Microbiol, 2019. **10**: p. 2006.
